# The complete chloroplast genome of halophyte *Kalidium foliatum* (Pall.) Moq., a dominant species of desert grassland

**DOI:** 10.1080/23802359.2022.2054379

**Published:** 2022-03-25

**Authors:** Jingru Wang, Shuang Wu, Minghao Wang

**Affiliations:** a School of Environmental Engineering, Xuzhou University of Technology, Xuzhou, China; b State Key Laboratory of Grassland Agro-Ecosystems, Institute of Innovation Ecology & School of Life Sciences, Lanzhou University, Lanzhou, China

**Keywords:** Complete chloroplast genome, halophyte, *Kalidium foliatum*, phylogenetic analysis

## Abstract

*Kalidium foliatum* (Pall.) Moq. is a dominant halophyte species of the desert ecosystem mainly distributed in Southeast Europe and Northwest Asia, and used as a major forage grass. Here, we report its complete chloroplast genome, assembled from the whole-genome resequencing data. The circular genome of *K. foliatum* is 153,773 bp in length, including a typical quadripartite structure consisting of a pair of inverted repeats (IRs; 24,991 bp) separated by large single-copy (LSC; 84,781 bp) and small single-copy (SSC; 19,010 bp) regions. The total GC content is 36.3%, and a total of 129 genes are annotated, including 84 protein-coding genes, 37 tRNAs, and eight rRNAs. The phylogenetic analysis has shown that *K. foliatum* is positioned as a sister taxon to the two *Salicornia* species, all belonging to the same tribe, Salicornieae.

*Kalidium foliatum* (Pall.) Moq.1849 is a small halophyte shrub with strong tolerance to saline-alkali soil and drought, which constitutes the main community in highly salinized areas. It is widely distributed throughout South-East European, Central and Southwest Asia, Russia (South Siberia), East China, Mongolia, and Kazakhstan. In Central Asia, *K. foliatum* is a succulent salt plant used as a major forage grass for horses, camels, and sheep, excellent for grazing herds in the winter (Liang and Wu 2017). Besides, it has been evidenced that the ethanol crude extracts of the aerial parts of *K. foliatum* have high antibacterial activity (Wang et al. 2015). To date, despite its importance, the chloroplast genome sequence of this species has not been reported.

In this study, we assembled and annotated the complete chloroplast genome sequence of *K. foliatum* based on whole-genome resequencing data. Fresh leaves were collected in Mazongshan Town, Jiuquan City, Gansu Province, China (41°48′ N, 97°02′ E), and dried and stored in silica gel. We received ethics approval for the sampling of the leaves of this plant from Qilian Mountain National Nature Reserve. The specimen was deposited at Lanzhou University (http://www.lzu.edu.cn/, Guangpeng Ren, rengp@lzu.edu.cn) under the voucher number QTP-LJQ-CHNM022-2010. The total genomic DNA was extracted using the modified CTAB procedure from the dried leaves (Doyle JJ and Doyle JL 1987), which was then sent to GenePlus-henzhen Clinical Laboratory for sequencing. A paired-end library (2 × 150 bp) was constructed and sequenced using the DNBSEQ-T7 Platform. The raw sequencing reads were first quality-checked by FASTQC (Andrews 2010), then the clean reads were used to de novo assemble the complete chloroplast genome using NOVOPlasty v3.8.3 (Dierckxsens et al. 2017). Subsequently, the chloroplast genome was annotated using GeSeq (Tillich et al. 2017) with MPI-MP chloroplast references and HMMER profile search and manually checked in Geneious v10.2.6 (Kearse et al. 2012). The chloroplast genome sequence has been deposited into the GenBank (accession number: OL397049).

The chloroplast genome of *K. foliatum* is a circular DNA molecule with 153,773 bp in length, including a typical quadripartite structure consisting of a pair of inverted repeats (IRs; 24,991 bp) separated by large single-copy (LSC; 84,781 bp) and small single-copy (SSC; 19,010 bp) regions. The total GC content of the genome is 36.3%. The genome harbors 129 genes, including 84 protein-coding genes (PCGs), 37 tRNAs, and eight rRNAs. Most genes occurred in single-copy, and 18 genes were duplicated in the IR regions: four rRNA genes (4.5S, 5S, 16S, and 23S rRNA), six PCGs (*rps19*, *rpl2*, *ycf2*, *ndhB*, *rps7*, *rps12_3’*), and seven tRNA genes (*trnI*-CAU, *trnL*-CAA, *trnV*-GAC, *trnI*-GAU, *trnA*-UGC, *trnR*-ACG, and *trnN*-GUU). The *rps12* gene was a unique trans-spliced gene with three exons. Of the annotated genes, 13 contained a single intron, whereas four had two introns.

To evaluate the phylogenetic relationship of *K. foliatum*, 12 Chenopodiaceae and one Amaranthaceae (as outgroup) chloroplast genomes were downloaded from GenBank and aligned with MAFFT v7.453 (Katoh and Standley 2013). The maximum-likelihood analysis was performed with IQTREE v2.1.3 (Nguyen et al. 2015) using the GTR + F+R2 substitution model and 100 bootstrap replicates to access node support. The result suggests that *K. foliatum* is clustered with the two *Salicornia* species in a strong supported clade (100%) (Figure 1). The molecular data confirm the taxonomic treatment that all the three species belong to the same tribe, Salicornieae. Our data provide new genetic resource for evolutionary and comparative genomics analyses with other Chenopodiaceae species in the future.

**Figure 1.**
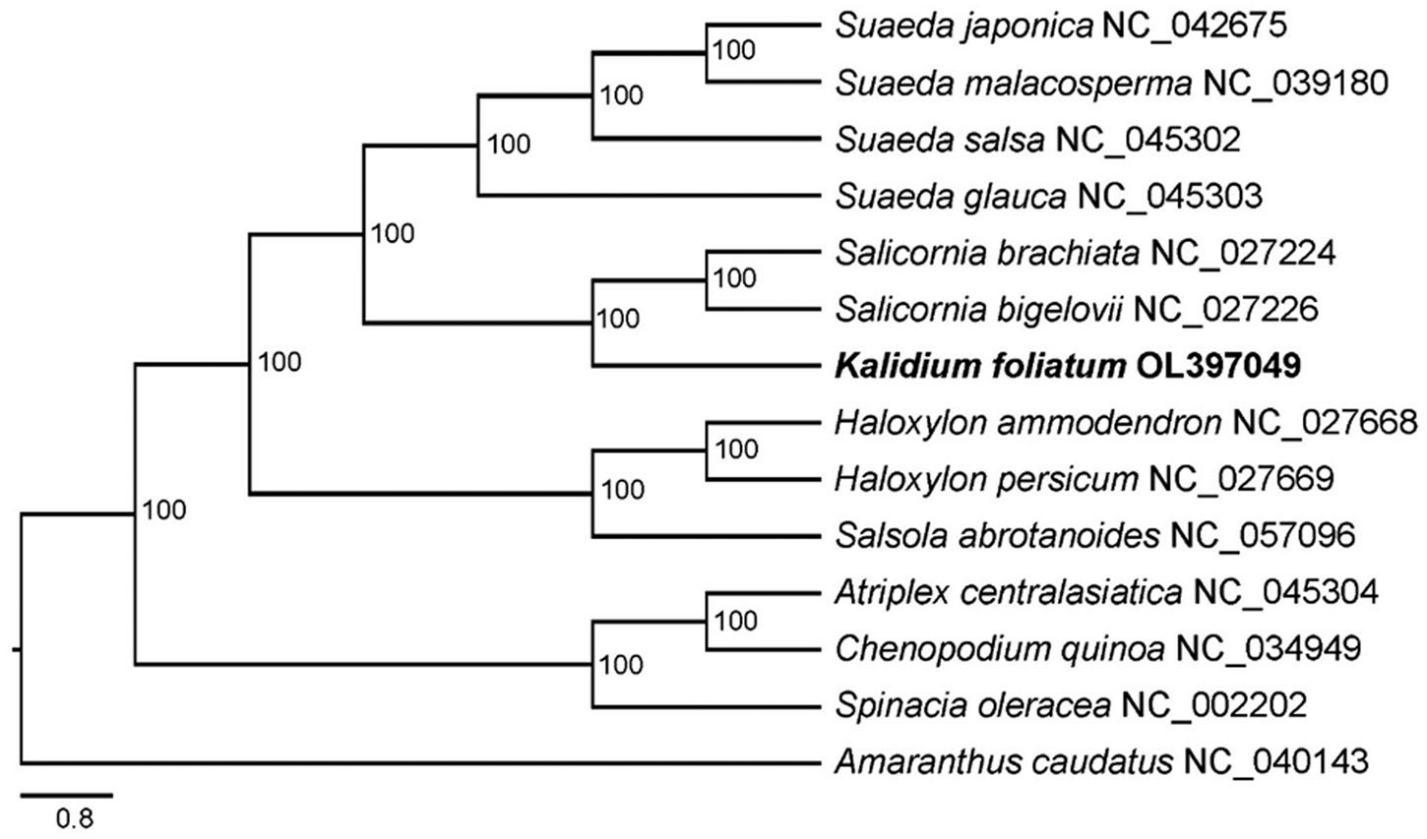
Phylogenetic tree inferred by maximum-likelihood (ML) method based on chloroplast genome sequences of *Kalidium foliatum* and other 13 species, with *Amaranthus caudatus* as outgroup. The bootstrap values based on 100 replicates are shown at the nodes. The species names are followed by their GenBank accession numbers.

## Data Availability

The data that support the findings of this study are openly available in GenBank of NCBI at https://www.ncbi.nlm.nih.gov/ under the accession no. OL397049. The associated BioProject, SRA, and Bio-Sample numbers are PRJNA778138, SRR16820175, and SAMN22929590, respectively.
